# Overcoming Xenoantigen Immunity to Enable Cellular Tracking and Gene Regulation with Immune-competent “NoGlow” Mice

**DOI:** 10.1158/2767-9764.CRC-24-0062

**Published:** 2024-04-09

**Authors:** Timothy N. Trotter, Andrea Wilson, Jason McBane, Carina E. Dagotto, Xiao-Yi Yang, Jun-Ping Wei, Gangjun Lei, Hannah Thrash, Joshua C. Snyder, Herbert Kim Lyerly, Zachary C. Hartman

**Affiliations:** 1 Department of Surgery, Duke University, Durham, North Carolina.; 2 Department of Pathology, Duke University, Durham, North Carolina.; 3 Department of Pharmacology and Cancer Biology, Duke University, Durham, North Carolina.; 4 Department of Cell Biology, Duke University, Durham, North Carolina.; 5 Department of Integrative Immunobiology, Duke University, Durham, North Carolina.

## Abstract

The ability to temporally regulate gene expression and track labeled cells makes animal models powerful biomedical tools. However, sudden expression of xenobiotic genes [e.g., GFP, luciferase (Luc), or rtTA3] can trigger inadvertent immunity that suppresses foreign protein expression or results in complete rejection of transplanted cells. Germline exposure to foreign antigens somewhat addresses these challenges; however, native fluorescence and bioluminescence abrogates the utility of reporter proteins and highly spatiotemporally restricted expression can lead to suboptimal xenoantigen tolerance. To overcome these unwanted immune responses and enable reliable cell tracking/gene regulation, we developed a novel mouse model that selectively expresses antigen-intact but nonfunctional forms of GFP and Luc, as well as rtTA3, after CRE-mediated recombination. Using tissue-specific CREs, we observed model and sex-based differences in immune tolerance to the encoded xenoantigens, illustrating the obstacles of tolerizing animals to foreign genes and validating the utility of these “NoGlow” mice to dissect mechanisms of central and peripheral tolerance. Critically, tissue unrestricted NoGlow mice possess no detectable background fluorescence or luminescence and exhibit limited adaptive immunity against encoded transgenic xenoantigens after vaccination. Moreover, we demonstrate that NoGlow mice allow tracking and tetracycline-inducible gene regulation of triple-transgenic cells expressing GFP/Luc/rtTA3, in contrast to transgene-negative immune-competent mice that eliminate these cells or prohibit metastatic seeding. Notably, this model enables *de novo* metastasis from orthotopically implanted, triple-transgenic tumor cells, despite high xenoantigen expression. Altogether, the NoGlow model provides a critical resource for *in vivo* studies across disciplines, including oncology, developmental biology, infectious disease, autoimmunity, and transplantation.

**Significance:**

Multitolerant NoGlow mice enable tracking and gene manipulation of transplanted tumor cells without immune-mediated rejection, thus providing a platform to investigate novel mechanisms of adaptive immunity related to metastasis, immunotherapy, and tolerance.

## Introduction

The fundamental function of the adaptive immune system is to discriminate between foreign and self-antigen–expressing cells via T- and B-cell responses to eliminate pathogens while leaving noninfected, self-antigen–expressing cells intact. This is accomplished by initial positive and negative selection of T-cell receptors (TCR) in the thymus, or B-cell receptors (BCR) in the bone marrow, with subsequent fine tuning in the periphery via a network of costimulatory molecules (1). Although tolerance to self-antigens is crucial for preventing autoimmunity, cancer presents a difficult challenge where the line between self and foreign antigens become blurred. Many immunotherapeutic strategies are based on the ability to elicit, or reinvigorate, antitumor T-cell repertoires that would otherwise be considered “self” or “near-self”, such as we have previously demonstrated with vaccines targeting receptor tyrosine-protein kinase erbB-2 (HER2) (2, 3). Ultimately, induction of self-antigen-specific immunity is critically related to the level of central and peripheral immune-tolerance to the specific antigen, which uniquely varies given its expression in thymic/bone marrow compartments (central tolerance), as well as throughout the body (peripheral tolerance). Thus, understanding antigen-specific tolerance is crucial for the success of immunotherapy and represents a critical hurdle, which has only been partially clinically overcome by systemic immune checkpoint blockade (3).

Immune tolerance to specific antigens is not only a conceptual hurdle in clinical immunotherapy, but is in fact a major challenge when attempting to accurately model human cancers in animals–especially in the context of tumor dissemination and metastasis (4–6). Recognition of these “passenger” antigens can lead to off-target selection and fundamentally skew the immune response, thereby altering study interpretation and potentially masking otherwise novel insights into tumor biology in clinically relevant, immune-competent hosts. Knowledge of this caveat is not new, and studies highlighting the immunologically based, deleterious effects of green fluorescent protein (GFP; used to track cells) have been reported for more than 20 years (4, 7). In addition to GFP, many tumor cell-centric studies rely upon tumor cells modified with foreign genes for selection (puromycin, hygromycin, neomycin, etc.) and manipulation of gene expression [reverse tetracycline-controlled transactivator (rtTA), Cas9, etc.], several of which have also been formally documented to elicit strong immune responses (6, 8–10). Very rarely is an antigen present in a *de novo* tumor that is wholly foreign to the immune system, and addition of xenobiotic genes grants the adaptive immune system an atypical advantage that severely limits the usefulness of syngeneic cell lines when investigating tumor–immune interactions. Thus, tolerant models are a pressing need for reliable experimentation. In addition, to track tumor progression *in vivo* and identify even small populations of disseminated tumor cells, protein tags, such as Firefly Luciferase (Luc) or GFP, must be utilized. Although others have previously tolerized animals to these proteins, background fluorescence or bioluminescence precludes the ability to differentiate between transplanted and host cells without additional staining steps (5, 11).

To overcome the obstacles presented here, and to address larger questions of immune tolerance, we developed a model (herein termed “NoGlow”) containing Cre-directed expression of rtTA as well as functionally dead GFP and Luc. Tissue-restricted expression of these genes renders NoGlow mice tolerant to GFP, Luc, and rtTA to varying degrees, depending on where the antigens are expressed, and do not reject transgenic tumor cells expressing any or all of these proteins. Moreover, tumor cells can be monitored via bioluminescence or immunofluorescence without background signal from the surrounding tissues and *de novo* metastasis can be observed after orthotopic implantation. Together, NoGlow mice provide a highly customizable tool for studying tumor–immune interactions at each stage of tumor progression and are amenable to broad questions of immune tolerance in multiple contexts, including autoimmunity, microbiology, transplantation, and gene therapy.

## Materials and Methods

### Mice

SCID-beige (C.B-Igh-1b/GbmsTac-Prkdcscid-Lystbg N7; model CBSCBG) and BALB/c (BALB/cAnNTac; model BALB) animals were purchased from Taconic Biosciences. Glowing Head (GH; C57BL/6-Tyrc-Brd Tg(Gh1-luc/EGFP)D8Mrln/J), CAG Luc-GFP (B6;FVB-Ptprca Tg(CAG-luc,-GFP)L2G85Chco Thy1a/J), doxycycline-inducible GFP (B6;129S4-Gt(ROSA)26Sortm1(rtTA*M2)Jae Col1a1tm7(tetO-HIST1H2BJ/GFP)Jae/J), FoxP3-GFP (B6.129(Cg)-Foxp3tm3(DTR/GFP)Ayr/J), CMV Cre (B6.C-Tg(CMV-cre)1Cgn/J), MMTV Cre (Tg(MMTV-cre)4Mam/J), pancreatic and duodenal homeobox 1 (Pdx1) Cre (B6.FVB-Tg(Pdx1-cre)6Tuv/J), and Probasin (Pbsn) Cre (FVB;B6-Tg(Pbsn-cre)20Fwan/J) mice were purchased from The Jackson Laboratory. WAP-HER2 (12) mice were kindly provided by Wei-Zen Wei (Wayne State University, Detroit, MI) and MMTV CAG-HER2 (13) mice were generously provided by Joshua Snyder (Duke University, Durham, NC). Genotyping and genetic monitoring for all strains were performed by Transnetyx. Details of each model used throughout the manuscript can be found in Supplementary Table S1. Embryonic stem (ES) cell targeting and injection was performed by the Duke Transgenic Mouse Core. pR26 CAG/BFP Dest was a gift from Ralf Kuehn (Addgene plasmid # 74282; http://n2t.net/addgene:74282; RRID:Addgene_74282; ref. 14). For a detailed description on transgenic model generation via ES cell targeting, please see ref. 15. The NoGlow^fl/fl^ (stock no. 039236) and constitutive NoGlow^+/+^ (stock no. 039259) mice have been deposited at The Jackson Laboratory.

### Cell Lines

E0771 cells were purchased from CH3 BioSystems (Amherst) and B16-F10 cells were obtained from the ATCC via the Duke Cell Culture Facility. Cells were authenticated by the respective manufacturer, low passage stocks were frozen upon acquisition, and cultured cells were monitored for changes in growth and morphology. Engineered cells were checked for construct expression via microscopy for GFP fluorescence. For all *in vivo* studies cells were used prior to passage 6. Cell lines were routinely tested and confirmed *Mycoplasma* negative via MycoStrip (InvivoGen) and were maintained in DMEM (Gibco) containing 10% FBS and penicillin (10 U/mL)/streptomycin (10 µg/mL) at 37°C and 5% CO_2_.

### Lentiviral Production and Infection

All lentiviral vectors were produced in 293T cells using second-generation packaging plasmids and previously described techniques, and viral stocks were concentrated by ultracentrifugation (16). Viral stocks were added to medium containing 8 µg/mL polybrene and cells were incubated for 48 hours before subculturing. Transduced cells were selected by sorting for GFP^+^ cells.

### Orthotopic Implantation

E0771 cells (10^6^) in sterile PBS were implanted into the fourth inguinal mammary fat pads of female mice and B16-F10 cells (10^5^) were implanted subcutaneously in the flank of male mice under general anesthesia. Tumors were monitored by caliper and volume was calculated using the formula: volume = (length × width^2^) ÷ 2.

### Adenoviral Production and Vaccination

Adenoviral vectors were generated and produced as previously described using Gateway cloning of ENTRY plasmid clones into pAd-CMV5 vectors (Invitrogen; ref. 2). Animals were vaccinated with 2.6 × 10^10^ viral particles of adenoviral vectors under isoflurane anesthesia via foot pad as before (2, 17).

### Bioluminescent/Fluorescent Animal Imaging

Tumor-bearing animals were injected intraperitoneally with d-luciferin (100 mg/kg) and live, whole-body bioluminescence intensity was measured using a LI-COR Pearl or IVIS Kinetic. Fluorescence in live mice was measured by an IVIS Kinetic. Lungs/livers were isolated for further ex vivo imaging immediately after euthanasia via CO_2_ inhalation.

### Serum ELISA

Immulon 4 HBX (Thermo Fisher Scientific) plates were coated with 1 µg/mL recombinant A. victoria GFP (Abcam ab84191) or P. pyralis Luciferase (Sigma SRE0045) at 4°C. The next day, plates were washed with PBS + 0.05% Tween 20 and blocked with PBS + 1% BSA (Sigma) for 1 hour at 37°C. A serial dilution (in 1% BSA/PBS) of serum was added in duplicate for 2 hours at room temperature followed by anti-mouse IgG streptavidin-HRP–conjugated antibody (1:2,000 in 1% BSA/PBS; Cell Signaling Technology) for 1 hour at 37°C. Plates were developed with TMB substrate (BioLegend), stopped with 0.18 mol/L H_2_SO_4_, and read at 450 nm on a Bio-Rad 680 microplate reader.

### Immunohistochemistry (IHC)

IHC staining on formalin-fixed, paraffin-embedded tissue sections was performed as before (18). Briefly, slides were deparaffinized and rehydrated, epitope retrieval was performed in a Retriever 2100 (Aptum Biologics) with R-Buffer A (Electron Microscopy Sciences), and endogenous peroxidases/phosphatases were quenched with BLOXALL blocking solution (Vector Laboratories). Tissues were blocked with Animal-Free Blocker R.T.U. (Vector Laboratories), probed with anti-Luciferase antibodies (Abcam; AB185923, 1:200 dilution) overnight at 4°C, washed with PBS, and incubated with ImmPRESS anti-Rabbit polymer detection reagent (Vector Laboratories) for 30 minutes at room temperature. Visualization was performed by incubation with 3,3′-diaminobenzidine (DAB; Vector Laboratories), tissues were counterstained with Gill No.3 Hematoxylin (Sigma), coverslipped, and imaged on an Olympus IX73 inverted microscope.

### Immunofluorescence (IF)

Deparaffinization, rehydration, and antigen retrieval of tissue sections was performed as with IHC. Tissues were blocked with Animal-Free Blocker R.T.U. for 30 minutes at room temperature followed by incubation with chicken IgY anti-GFP antibodies (Aves Labs; GFP-1020, 1:250 dilution) overnight at 4°C. Tissues were washed with PBS, and secondary staining was performed for 1 hour in the dark at room temperature with Donkey anti-chicken IgY AF488–conjugated antibodies (Jackson Immunoresearch; 703–545–155, 1:1,000). Tissues were counterstained with DAPI, coverslipped with VECTASHIELD Vibrance (Vector) mounting media, and imaged on a Zeiss LSM 880 confocal microscope.

### Immunocytochemistry

293T cells were transduced with Adenoviral vectors in 8-well chamber slides (NEST Scientific 1162B43). After 24 hours, cells were fixed with 10% formalin, washed with PBS, blocked/permeabilized in Animal-Free Blocker containing 0.3% Triton X-100, and incubated overnight with Rabbit anti-Luciferase (Abcam; ab185923, 1:500 dilution) or Chicken anti-GFP antibodies (Aves Labs; GFP-1020, 1:500 dilution) at 4°C. The next day, wells were washed, secondary staining was performed with goat anti-rabbit AF594 (Invitrogen; A-11012, 1:1,000) or donkey anti-chicken AF488 (Jackson Immunoresearch; 703–545–155, 1:1,000) conjugated antibodies, counterstained with DAPI, mounted with VECTASHIELD, and imaged on an Olympus IX73 inverted microscope.

### ELISpot

Mouse splenocytes were harvested by passing whole spleens through a 70-µm filter and red blood cells were lysed with ACK buffer. IFNγ ELISPOT assays (Mabtech Inc.) were performed according to the manufacturer's instructions. Briefly, splenocytes (500,000 cells/well) were stimulated for 18 hours with the indicated peptides at 1 µg/mL in complete RPMI1640 medium. PMA (50 ng/mL) and ionomycin (1 µg/mL) (Sigma) were used as positive controls. For a complete list of peptide sequences, see Supplementary Table S2.

### Luciferase Activity and Western Blot Analysis

293T cells were transfected (PEI) with plasmid (0.2 µg DNA/2 × 10^4^ cells) and collected after 24 hours for luciferase activity on a Veritas Microplate Luminometer (Turner Biosystems). For Western blots, 4%–15% Mini-PROTEAN TGX Stain-Free Gels (Bio-Rad) were run with 30 µg protein lysate, transferred to Immoblin-FL PVDF membranes (Millipore), blocked with Intercept (PBS) blocking buffer (LI-COR), and stained with anti-Luciferase antibodies (Abcam; AB185923, 1:1,000 dilution) in blocking buffer overnight at 4°C with rocking. Membranes were then washed with PBS, secondary staining was performed with IRDye 800CW Goat anti-Rabbit IgG secondary antibody (LI-COR; 926–32211; 1:2,000) at room temperature for 1 hour. Gels were then washed, blocked, incubated with mouse anti–β-actin (Cell Signaling Technology; 8H10D10, 1:1,000) and IRDye 680RD goat anti-mouse IgG secondary antibody (LI-COR; 926–68070, 1:2,000) for 30 minutes at room temperature, and the blot was visualized on a LI-COR Odyssey.

### Study Approval

All animal studies were performed in accordance with Duke IACUC approval (protocol A043–23–02) and supervised/housed by the Division of Laboratory Animal Resources (DLAR). For studies, mice were randomized and we did not observe any attrition of mice due to death or other factors, thus no mice were excluded from analysis. All mice utilized in these studies were adults from 6 weeks to 16 weeks of age, weighing more than 20 g. Replicates are noted in figure legends.

### Statistics and Data Availability

Data were visualized and analyzed using GraphPad Prism v10. Details of statistical analyses can be found in the figure legends and *P* values are displayed within the figures. *P* values of 0.05 or less were considered significant. The data generated in this study and detailed protocols are available upon request from the corresponding author.

## Results

### Tolerance to Foreign Genes Heavily Relies on the Specific Animal Model

**FIGURE 1:**
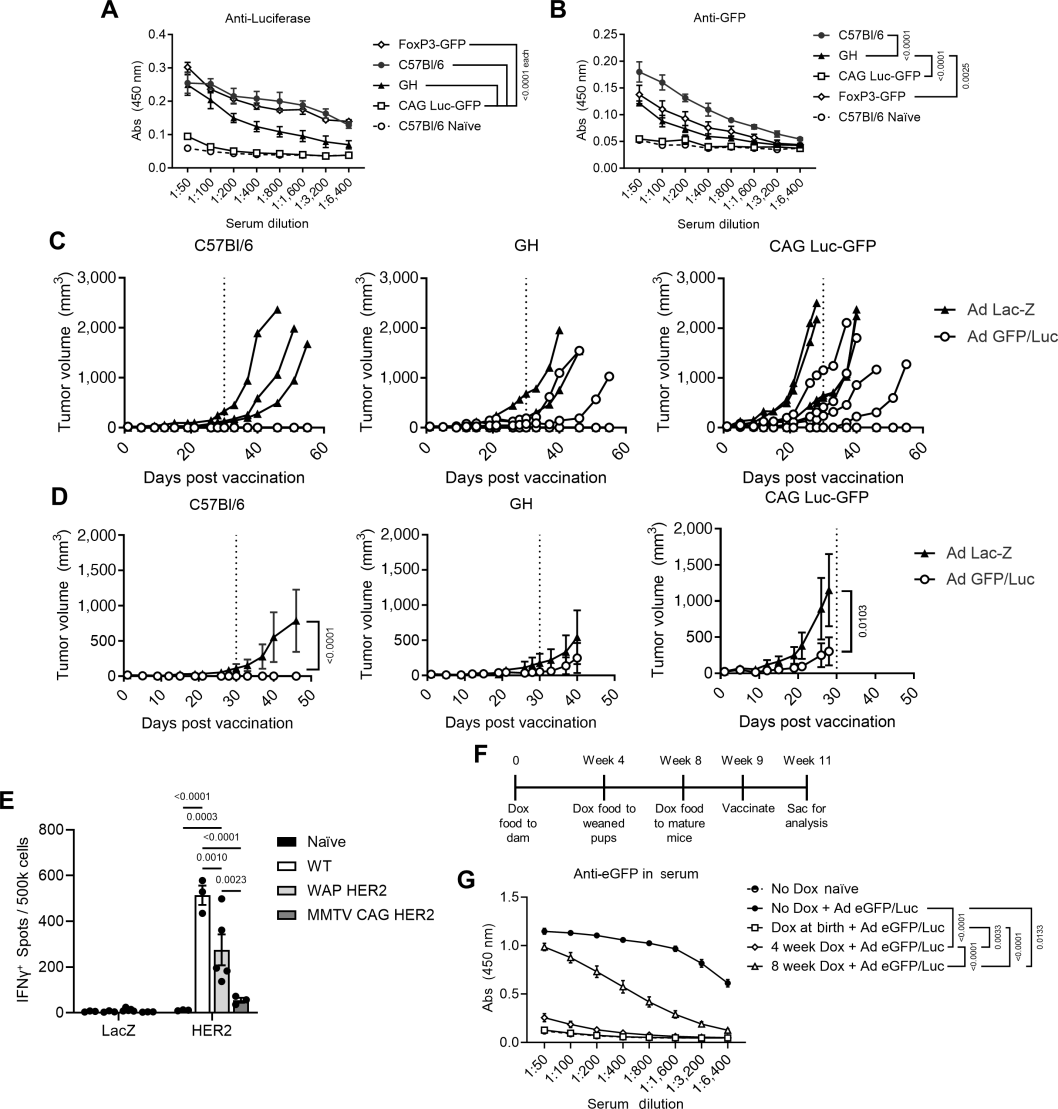
Tolerance to foreign antigens is animal model specific. **A** and **B,** Mice expressing GFP and Luc in the pituitary (GH; *n* = 5), full-body GFP, and Luc (CAG Luc-GFP; *n* = 5), in FoxP3^+^ cells (Foxp3-GFP; *n* = 4), or wild-type (WT) C57Bl/6 (*n* = 4) were vaccinated with adenovirus (Ad) encoding GFP/Luc (2.6 × 10^10^ vp/mouse) and anti-Luciferase (A) or anti-GFP (B) in the serum were evaluated by ELISA after 2 weeks. Only CAG Luc-GFP animals, which express both GFP and Luc, display humoral tolerance to both upon vaccination. *P* values shown are at 1:50 dilution by two-way ANOVA with Tukey multiple comparisons test. **C** and **D,** E0771 GFP-Luc cells were implanted into the MFP of female WT C57Bl/6, GH, or CAG Luc-GFP mice (*n* = 10 each) and subsequently vaccinated with Ad-GFP/Luc or Ad-LacZ as control on day 3 (*n* = 5/group each). Growth of individual tumors for the duration of the experiment (C) or average tumor volume until time of first euthanasia (D) comparing control or antigen-specific Ad demonstrate that E0771 GFP-Luc cells grow significantly larger in tolerant CAG Luc-GFP mice. *P* values shown are at day of first euthanasia by two-way ANOVA with Bonferroni multiple comparisons test. **E,** Female WT (*n* = 3) or mice expressing human HER2 under the whey acid protein (WAP) promoter (WAP HER2; *n* = 5) or MMTV Cre–driven CAG promoter (MMTV CAG HER2; *n* = 3) were vaccinated with Ad-Human Her2 and splenocytes were analyzed for anti-HER2 responses by ELISPOT after 3 weeks. WAP HER2 mice displayed reduced anti-HER2 responses compared with WT animals; however, MMTV CAG HER2 animals produced a limited anti-HER2 T-cell response compared with both WT and WAP HER2. Groups were compared by two-way ANOVA with Tukey multiple comparisons test. **F,** Doxycycline-inducible GFP animals were given doxycycline (dox)-containing chow at the indicated time points, vaccinated at 9 weeks old, and euthanized after two weeks. **G,** Serum anti-GFP responses in animals from F reveal that GFP remains highly immunogenic in mice with late (8 weeks old) versus early (4 weeks old/at birth) expression of GFP via doxycycline. *P* values shown are at 1:50 dilution by two-way ANOVA with Tukey *post hoc* analysis. All *P* values represent mean ± SEM.

In our previous studies, we obtained Glowing Head (GH) mice, which express GFP and Luc in the mouse pituitary (11), to track disseminated tumor cells. We initially observed a lack of tolerance in pilot experiments, therefore we formally assessed the degree of immune tolerance in this model via antigen-specific vaccination. Using Adenoviral vectors encoding GFP and Luc, GH mice were compared to Foxp3-GFP mice (eGFP expression restricted to Foxp3+ cells), CAG Luc-GFP mice (ostensibly fully tolerant mice that constitutively express GFP and Luc under a CAG promoter), or wild-type (WT) C57Bl/6 animals. As expected, CAG Luc-GFP demonstrated negligible antibody response to Luc (Fig. 1A) or GFP (Fig. 1B) and appeared to be highly tolerant to these antigens. Compared with WT, GH mice were somewhat tolerant to GFP and Luc (Fig. 1A and B), with tolerance to GFP equivalent to the restricted Foxp3-GFP mice (Fig. 1B). We next tested whether the limited tolerance observed via vaccination impacted tumor engraftment by implanting stable GFP and Luc-expressing, syngeneic E0771 mammary tumor cells into the mammary fat pad (MFP) of GH animals. We previously demonstrated that E0771 cells contain a high mutational burden and elicit a robust immune response in line with human triple-negative breast cancer (TNBC), thus an ideal model for tumor immunologic studies (19). While orthotopic tumors formed in GH mice, growth kinetics were highly variable and did not significantly differ from WT mice. In contrast, CAG Luc-GFP mice formed significantly larger tumors than either GH or WT mice (Supplementary Fig. S1A). The limits of tolerance were further tested with Ad-GFP/Luc vaccination of WT, GH, or CAG Luc-GFP three days after implanting E0771 GFP-Luc cells into the MFP of female mice. This therapeutic vaccination completely prevented tumor outgrowth in WT animals but did not result in a benefit over control vaccination in GH animals (Fig. 1C). Interestingly, vaccination was still able to delay tumor growth in CAG Luc-GFP animals (Fig. 1D), although these tumors grew significantly larger than Ad-GFP/Luc vaccinated WT or GH mice (Supplementary Fig. S1B). These data illustrate that while tolerance can be partially broken to widely expressed proteins, preclinical vaccine studies will be greatly influenced by the overall degree of tolerance to transplanted tumors.

Because the former experiments were performed in animals expressing the xenoantigen in substantially different tissues and cell types, we next investigated tolerance when the same antigen was largely restricted to the same tissue by different promoters. Both mouse mammary tumor virus (MMTV) and whey acidic protein (WAP) have been extensively used to study mammary gland biology and for endogenous mammary tumor models (20–23). Human HER2 (hHER2) expression under the WAP promoter (WAP-HER2) was also shown to tolerize animals to this foreign gene (12). Therefore, Balb/c WAP-HER2 mice were vaccinated against full-length hHER2 and compared with Balb/c animals expressing CAG-driven, floxed hHER2 in the presence of MMTV-Cre (MMTV CAG-HER2; ref. 13). Similar to previous reports, female WAP-HER2 mice were indeed more tolerant to hHER2 compared to WT Balb/c animals (12); however, MMTV CAG-HER2 mice were significantly more tolerized to hHER2 with limited reactive T cells in the spleen (Fig. 1E). MMTV CAG-HER2 mice also displayed similar levels of tolerance to hHER2 on a C57Bl/6 background (Supplementary Fig. S1C). These data further indicate that the specific promoter complex has a significant downstream impact on overall tolerance to foreign genes, even when the target tissue is the same, perhaps due to differences in generating central tolerance.

Central tolerance is dictated by coordinated expression of self-antigens in the thymus (T cells) or bone marrow (B cells) during development, and as mice age so does the ability to form central tolerance (24). Whether as endogenous inducible models, or implantation of syngeneic tumor cell lines, foreign antigens for tumor identification are typically introduced in adulthood after central tolerance is established. Therefore, we investigated if delayed induction of a foreign reporter impacts tolerance to that reporter with doxycycline-inducible eGFP mice. Animals were provided with doxycycline chow either early during development via the nursing dam, at weening (4 weeks old), or as adults (8 weeks), and all vaccinated with an Ad-eGFP/Luc at 9 weeks of age (Fig. 1F). As expected, mice given doxycycline at birth were completely tolerant to eGFP, with mice given doxycycline at 4 weeks showing subtle but nonsignificant increases in anti-GFP serum responses (Fig. 1G). However, by 8 weeks tolerance was significantly reduced compared with earlier administration, although these animals were indeed more tolerant than control littermates with no doxycycline administration (Fig. 1G). Altogether, these data confirm that timing of foreign, even endogenously expressed, genes has a significant impact on overall tolerance in animal models that could bias antitumor immunity, particularly in early lesions.

### Mutant Forms of GFP and Luciferase Induce Protective Immunity in Mice

The totality of our initial investigations revealed that control of both timing and tissue restriction of xenoantigen exposure are fundamentally important components of tolerant tumor models. While mice can be tolerized to foreign reporter genes, native forms of fluorescent proteins or Luc in nontumor tissues limit their usefulness as tumor-specific reporters (5). Thus, we surmised that mutant, inactive forms of GFP and Luc could be used to maintain the reporter function of WT forms in transplantable cell lines. We initially validated inactivity of a reported eGFP mutant (ΔT64, corresponding to Thr63 in avGFP) after transduction of 293T cells with Adenovirus encoding either WT or mutant eGFP (25). While no native fluorescence was observed, mutant eGFP was detectable after antibody staining (Supplementary Fig. S2A). Similarly, dramatically reduced activity was observed in several previously reported Luc mutants after plasmid transfection (Supplementary Fig. S2B; refs. 26–28).

**FIGURE 2:**
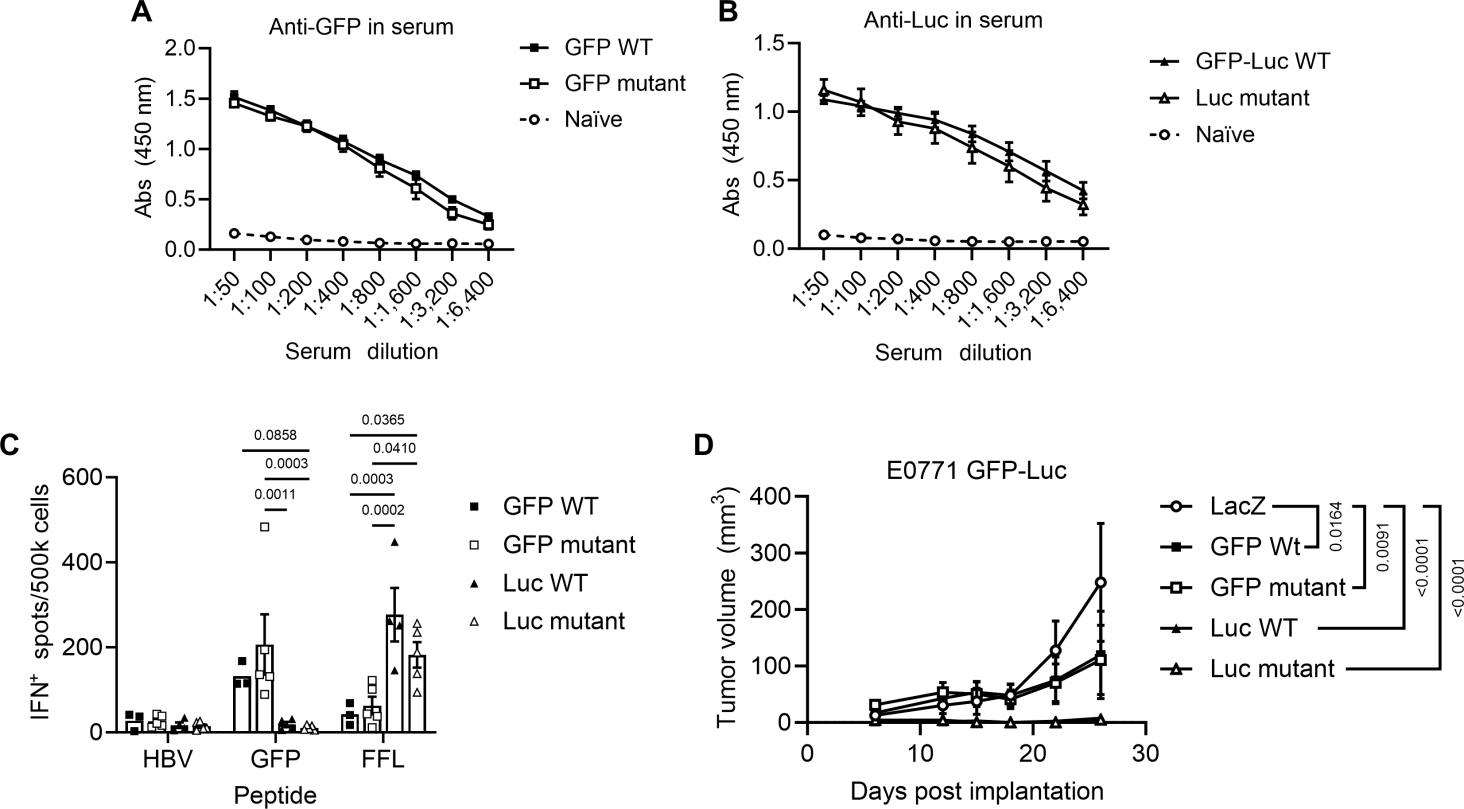
Mutant GFP and luciferase induce immune responses comparable with wild-type (WT) proteins. **A,** WT male Balb/c mice were vaccinated with Ad encoding WT or mutant copies of GFP (ΔT64; *n* = 5/group) and serum antibody responses were measured after 3 weeks. Mice showed equivalent anti-GFP responses whether vaccinated with WT or mutant GFP. **B,** WT female C57Bl/6 mice were vaccinated with Ad-GFP-Luc WT or Ad-Luc mutant (G315A; *n* = 5/group) and serum antibody responses were measured after 3 weeks revealing similar levels of anti-Luc antibodies. *P* values for A, B are at 1:50 dilution by two-way ANOVA with Tukey multiple comparisons test. **C,** Male WT Balb/c mice were vaccinated with Ad encoding WT or mutant GFP (ΔT64) or Luc (H245R) and anti-GFP or -Luc T-cell responses in the spleens were detected after 3 weeks via ELISpot. Both WT and mutant forms of GFP and Luc were able to elicit robust T-cell responses compared with mice naïve for the respective antigen. *P* values were determined by two-way ANOVA with Tukey *post hoc* analysis. **D,** E0771 GFP-Luc cells were implanted in MFPs of female WT C57Bl/6 mice and were vaccinated with Ad encoding WT GFP, mutant GFP (ΔT64), WT Luc, mutant Luc (ΔH245), or LacZ control (*n* = 5 per group) after three days. Compared with LacZ control vaccination, GFP WT/mutant, or Luc WT/mutant vaccines resulted in a comparable reduction in tumor growth. *P* values are at time of euthanasia by two-way ANOVA with Tukey multiple comparisons test. All *P* values represent mean ± SEM.

Next, the ability of mutant forms of GFP and Luc to elicit both B- and T-cell immunity were examined. Mice were vaccinated with Adenovirus encoding either WT or mutant forms of GFP and Luc and analyzed for antigen-specific antibody responses in the serum by ELISA or T cell responses in the spleen by ELISpot. Importantly, mutant forms of both GFP (Fig. 2A) and Luc (Fig. 2B) elicited vaccine-specific B-cell responses as well as T-cell responses (Fig. 2C) comparable with WT forms. Finally, to determine whether Ad-mediated, antimutant GFP and Luc responses provided protective antitumor immunity, E0771 GFP-Luc cells were implanted into the MFP of female FoxP3-GFP mice. By the end of the experiment, tumor outgrowth was largely prevented by vaccination against WT and mutant Luc compared with Ad-LacZ control (Fig. 2D). In addition, Ad-GFP-WT or -GFP mutant resulted in an equivalent reduction in tumor burden compared with control vaccination, although mice were somewhat protected by the GFP in Foxp3 cells (Figs. 1B and 2D). Together, these data demonstrate that mutant, inactive forms of GFP and Luc are similarly immunogenic in mice as WT forms. Furthermore, the tolerogenic protection against mutant GFP in Foxp3-GFP mice suggests immunologic interchangeability and supports the utility of nonfunctional proteins to generate tolerant animals.

### Generation of Triple-transgenic “NoGlow” Mice

**FIGURE 3:**
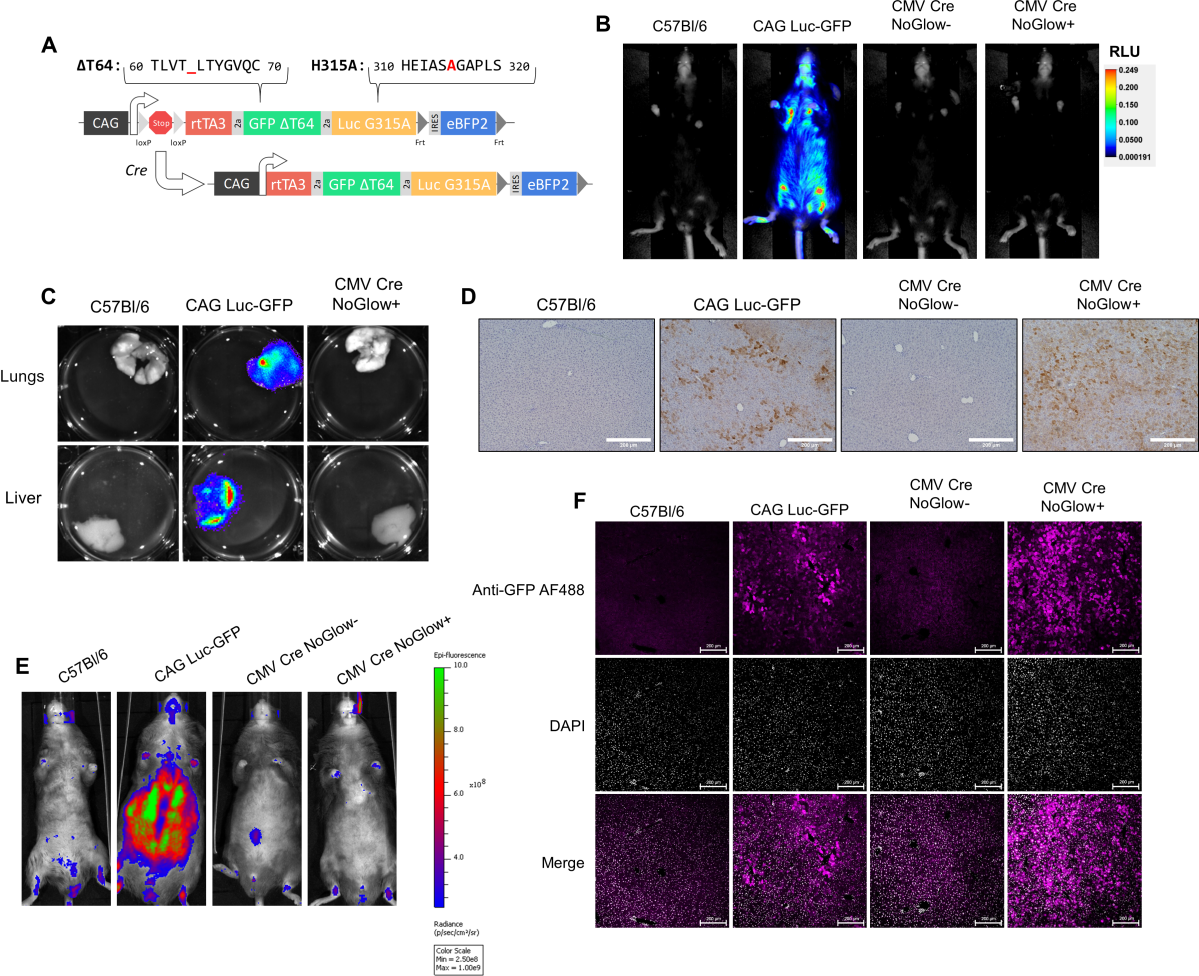
NoGlow mice express eGFP and Luciferase without background fluorescence and bioluminescence. **A,** Diagram of the NoGlow construct. LoxP-flanked stop site prevents expression of the NoGlow construct in the absence of Cre recombinase. However, in the presence of Cre the stop site is recombined to yield a single transcript encoding rtTA3 and mutant GFP/Luc driven by the CAG promoter. **B,** Chimeric founder NoGlow animals were crossed to CMV (full-body) Cre animals to test activity and expression of the construct. Representative bioluminescence imaging of female C57Bl/6, CAG Luc-GFP, and F1 CMV Cre NoGlow littermates with or without the NoGlow construct reveal no background bioluminescence in CMV Cre NoGlow+ animals. **C,** Bioluminescent imaging of representative lungs/livers shows no background bioluminescence in NoGlow+ mice. **D,** Chromogenic Luciferase staining in livers from NoGlow+ and control mice confirms luciferase protein expression despite the lack of bioluminescence in B and C. **E,** Representative fluorescent imaging of male C57Bl/6, CAG Luc-GFP, and F1 CMV Cre NoGlow animals shows no background fluorescence in CMV Cre NoGlow+ mice, while immunofluorescent staining (**F**) for GFP in liver sections from CMV Cre NoGlow+ animals confirms that GFP protein can be detected upon antibody staining.

To generate a tolerant mouse model that allowed engraftment, tracking, and temporal manipulation of gene expression in transplanted cells, we modified a ROSA26-targeted, CAG-driven vector (14) to express rtTA3, mutant GFP (ΔT64), and mutant Luc (G315A; the lowest signal of tested mutants) on a single transcript separated by 2A ribosomal skipping peptides (Fig. 3A; ref. 29). This construct contains an upstream loxP flanked stop cassette for Cre-mediated, tissue-restricted expression. Transfection of 293T cells with or without a secondary plasmid containing Cre recombinase demonstrated little background Luc expression (last in the transcript) in the absence of Cre (Supplementary Fig. S3A) and verified enzymatically dead Luc (Supplementary Fig. S3B). Successfully targeted ES cells were transplanted into pseudopregnant females to generate chimeric, transgene-positive mice.

For initial testing, we wanted to ensure central and peripheral, B- and T-cell tolerance. Thus, we opted to use the CMV-Cre model, which is turned on early during embryogenesis and results in widespread expression throughout the body including germ cells and cells in the thymus (T-cell tolerance) and bone marrow (B-cell tolerance; refs. 30–32). Therefore, NoGlow+ chimeras were subsequently crossed to CMV Cre animals for heterozygous NoGlow+ or WT NoGlow− littermates. No bioluminescence was detected upon full-body imaging of NoGlow+ mice (Fig. 3B) with evaluation of individual livers and lungs confirming these results (Fig. 3C). However, IHC staining for Luc protein in livers demonstrated robust expression in CMV Cre+ NoGlow+ animals (Fig. 3D). Similarly, no fluorescence was observed in CMV Cre NoGlow+ mice upon whole body fluorescence imaging (Fig. 3E) but subsequent IF staining of livers verified GFP expression in CMV Cre+ NoGlow+ mice (Fig. 3F).

### The NoGlow Model Exposes Cre-specific Variability in Immune Tolerance

**FIGURE 4:**
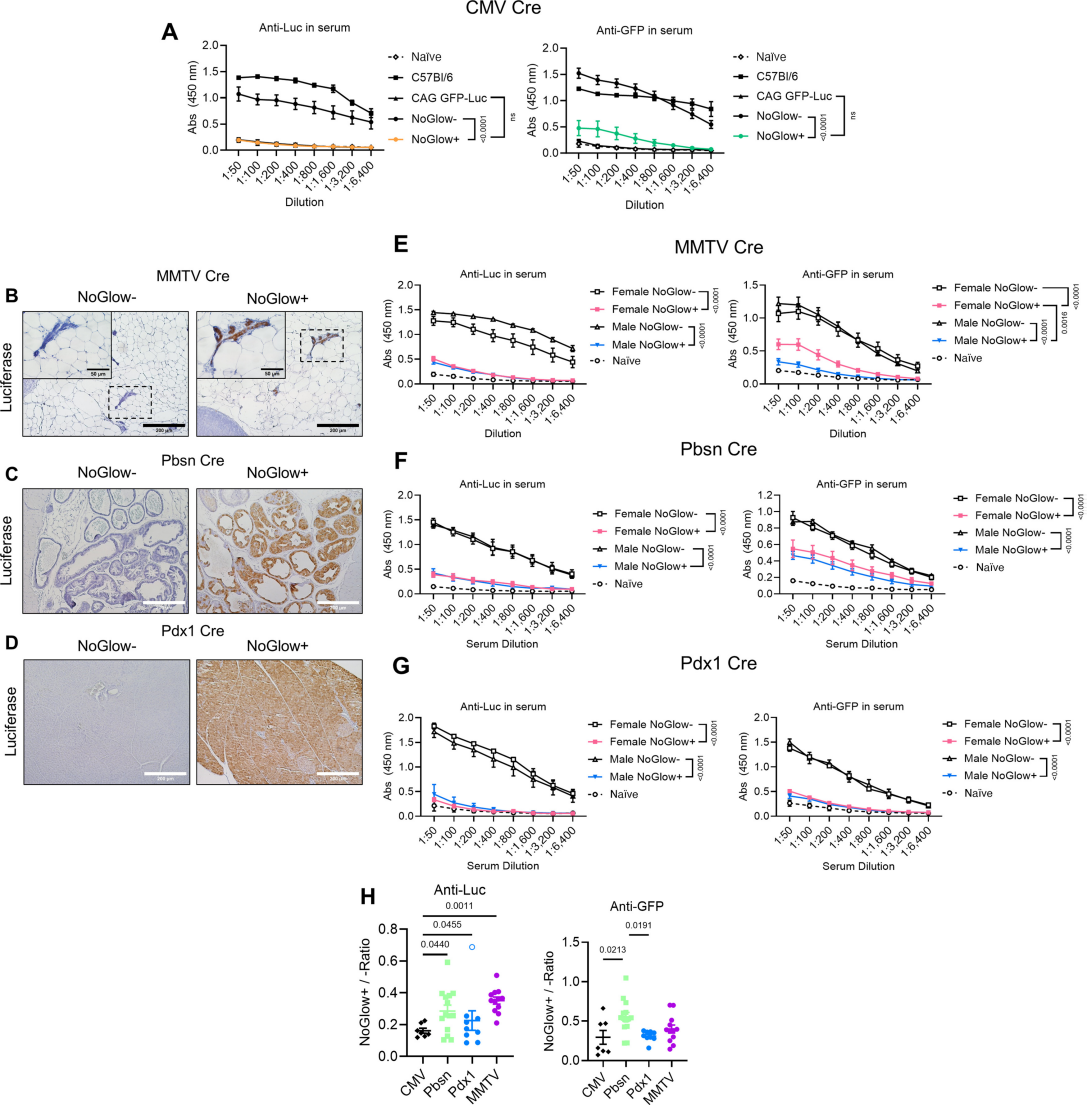
NoGlow mice reveal the impact of sex and tissue distribution on immune tolerance. **A,** Male WT (*n* = 3), CAG Luc-GFP (*n* = 3), and NoGlow+ (*n* = 6) or NoGlow− (*n* = 10) littermates were vaccinated with an Ad encoding WT GFP-Luc and serum was collected after two weeks for anti-Luc or anti-GFP responses. Unvaccinated naïve WT (*n* = 2) animals were used as baseline controls. **B,** Luc staining showing protein expression in mammary epithelium of female MMTV Cre NoGlow+ or NoGlow− littermates. **C,** Luc staining showing protein expression in prostate epithelium of male Pbsn Cre NoGlow+ or NoGlow− littermates. **D,** Luc staining demonstrating expression in acini and islets in pancreases of male Pdx1 Cre NoGlow+ or NoGlow− littermates. **E–G,** MMTV Cre (E; *n* = 4 NoGlow- F, *n* = 5 NoGlow+ F, *n* = 2 NoGlow- M, *n* = 7 NoGlow+ M, *n* = 3 naïve), Pbsn Cre (F; *n* = 6 NoGlow- F, *n* = 6 NoGlow+ F, *n* = 4 NoGlow- M, *n* = 8 NoGlow+ M, *n* = 1 naïve), or Pdx1 Cre (G; *n* = 2 NoGlow- F, *n* = 4 NoGlow+ F, *n* = 4 NoGlow- M, *n* = 5 NoGlow+ M, *n* = 2 naïve) littermates were vaccinated with an Ad encoding WT eGFP and Luc. Serum antibody responses to Luc and GFP were determined by ELISA two weeks postvaccination. *P* values for A, E, F, G are displayed at 1:50 and determined by two-way ANOVA with Tukey correction. **H,** Normalized ratio of serum anti-Luc or -GFP antibody responses in independent NoGlow+ versus mean of NoGlow− littermates across two independent experiments demonstrating highest tolerance in CMV Cre and Pdx1 Cre animals. *P* values were determined by one-way ANOVA with Tukey correction. All *P* values represent mean ± SEM.

Having validated expression from CMV whole body NoGlow mice, we next examined the impact of antigen-specific, vaccine-induced immune responses in CMV Cre NoGlow animals. Heterozygous CMV Cre NoGlow+ or NoGlow− littermates, CAG Luc-GFP, and WT C57Bl/6 animals were vaccinated with Ad-GFP/Luc and serum antibody responses were examined by ELISA. These data revealed that while robust anti-GFP or -Luc responses were detected in NoGlow− littermates, CMV Cre NoGlow+ animals did not significantly differ from CAG Luc-GFP or naïve mice (Fig. 4A). Thus, at least when widely expressed, NoGlow+ mice appear to be maximally tolerant to the transgenic xenoantigens.

Based upon the earlier finding that tissue restriction and/or Cre selection strongly influenced germline tolerance, NoGlow animals were also crossed to multiple Cre strains to confirm tissue specificity of the construct and overall tolerance in each line. We chose MMTV-Cre and Pbsn-Cre as semi-sex–dependent models, and Pdx1-Cre was used as an alternative to the rat insulin I promoter (RIP) that is widely reported to not induce central tolerance in mouse models (33, 34). Staining for Luc by IHC indicated transgene expression in the expected tissues (Fig. 4B–D). Interestingly, upon Ad-GFP/Luc vaccination, tolerance to the transgene was detected in both male and female MMTV- and Pbsn-Cre animals, although to a lesser degree in female than male MMTV-Cre mice (Fig. 4E and F). Likewise, in contrast to RIP-Cre reports, Pdx1-Cre mice were almost completely tolerant to GFP and Luc (Fig. 4G). Ultimately, when comparing NoGlow+ to NoGlow− littermates across Cres, the widely-expressed CMV-Cre animals were most tolerant with Pdx1-Cre showing similar results. Furthermore, MMTV- and Pbsn-Cre were overall significantly less tolerant compared with littermates (Fig. 4H). Together, these data reveal somewhat unexpected findings about spatially-restricted tolerance, particularly in the case of Pdx1-Cre, which supports our previous observations (Fig. 1) of tissue restricted, promoter driven tolerance across different models. In addition, these data demonstrate the utility of NoGlow mice for general investigations of tissue-specific peripheral tolerance and immunity.

### NoGlow Mice Permit Engraftment and *De Novo* Metastasis of Triple-transgenic Tumor Cells

**FIGURE 5:**
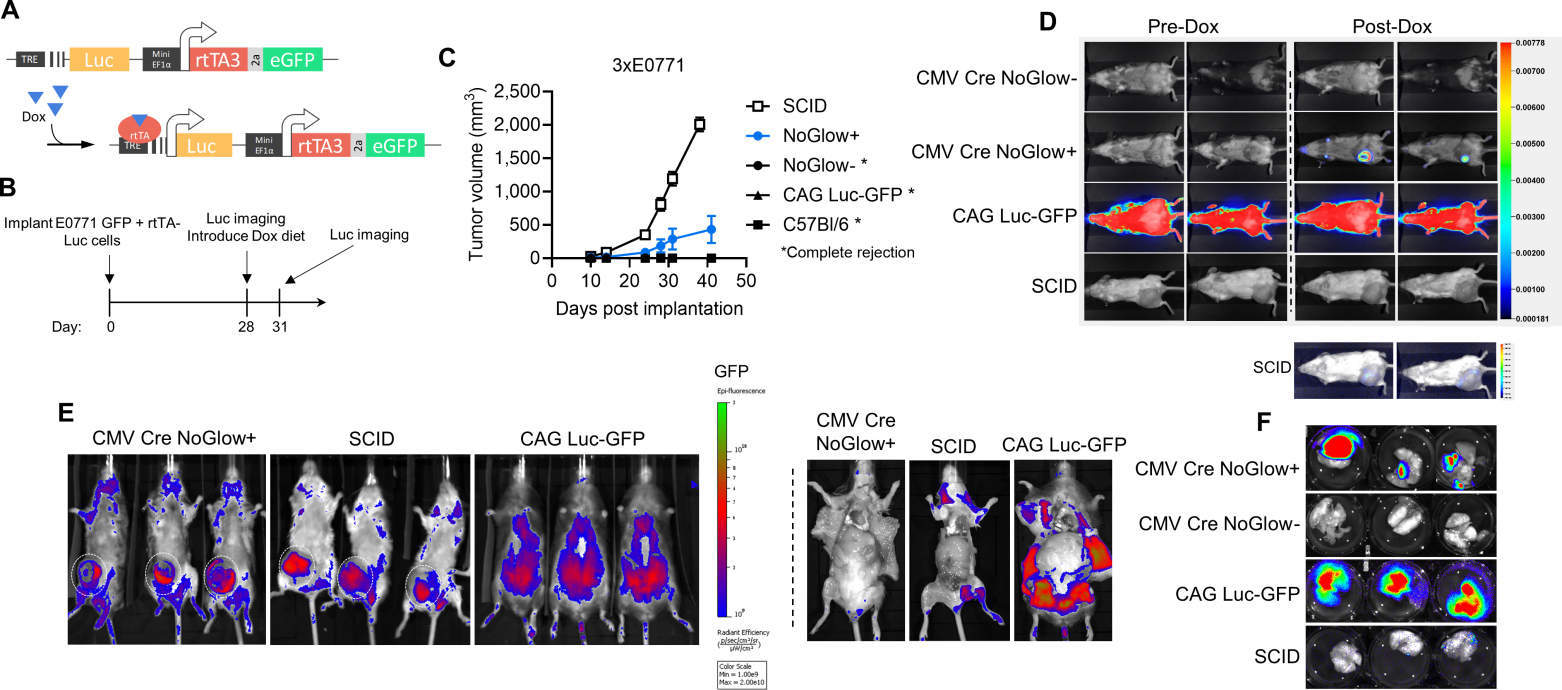
Tumor cells expressing GFP, rtTA, and Luciferase successfully engraft in NoGlow mice. **A,** Diagram of triple-transgenic (3 ×) E0771 cells. GFP and rtTA are constitutively expressed and Luc is induced with the addition of doxycycline. **B,** 3 × E0771 (10^6^) were implanted into the mammary fat pad (MFP) of wild-type (WT) C57Bl/6 (*n* = 7), CAG-driven Full-body WT GFP/Luc expressing mice (*n* = 7), CMV cre littermates positive (*n* = 6) or negative (*n* = 7) for the NoGlow construct, or SCID beige (*n* = 4) mice. **C,** Overall (top) and individual (bottom) tumor growth in each group. No evidence of tumors was observed in Full-body GFP-Luc, NoGlow−, or C57Bl/6 animals by the end of experiment. **D,** Representative bioluminescence imaging of animals pre (left) or post (right) doxycycline diet introduction. Bottom, Luc was induced in SCID mice but to a lesser degree compared with NoGlow+ mice. **E,** Representative GFP imaging upon euthanasia of Noglow+, SCID, and Full-body GFP-Luc mice. Left, concentrated GFP signal in tumors of NoGlow+ and SCID compared with Full-body GFP-Luc mice. Right, visceral GFP signal only in Full-body GFP-Luc mice. **F,** Representative lungs of NoGlow+, NoGlow−, Full-body GFP-Luc, or SCID mice upon euthanasia. Lung metastasis can be visualized in NoGlow+ and SCID animals.

After validation of strong antigen-specific immune tolerance without background fluorescence and luminescence in whole body NoGlow mice, we next tested their usefulness for monitoring tumor progression *in vivo*. In these studies, we tested the engraftment, temporal gene regulation, and metastasis of triple-transgenic E0771 (3xE0771) cells that constitutively express eGFP and rtTA separated by a 2a peptide, with the addition of a doxycycline-inducible Luc (Fig. 5A; Supplementary Fig. S4). Tumor cells were implanted into the MFP of heterozygous CMV Cre NoGlow+ or NoGlow− littermates, along with CAG Luc-GFP (no rtTA), WT C57Bl/6 (no eGFP, Luc, or rtTA), and SCID-beige mice to represent various degrees of tolerance to the xenoantigens present in 3xE0771 cells. In addition, mice were supplemented with Dox-containing chow on day 28 to turn on Luc expression in 3xE0771 tumors (Fig. 5B). To our surprise, all tumors were completely rejected in NoGlow- littermates, WT, and CAG Luc-GFP animals. Furthermore, while tumors were expectedly larger in SCID mice by the end of the experiment, 3xE0771 cells indeed formed orthotopic tumors in NoGlow+ mice (Fig. 5C). Importantly, Luc expression was detectable via bioluminescence in NoGlow+ mice indicating maintenance of all three xenoantigens (as Luc could only be induced if rtTA was present, and rtTA was translationally linked to eGFP; Fig. 5D). Upon euthanasia, whole-body imaging confirmed eGFP fluorescence was restricted to orthotopic 3xE0771 tumors and not observed in visceral organs of NoGlow+ animals (Fig. 5E). Moreover, bioluminescent imaging identified *de novo* metastases in both NoGlow+ and SCID animals (Fig. 5F), with NoGlow animals possibly showing higher metastatic burden in the lungs despite smaller primary tumors. Of note, even if residual tumor cells in the MFP or disseminated tumor cells were present in CAG Luc-GFP mice they would be undetectable by bioluminescent/fluorescent imaging (Fig 5D–F). Taken together, these data demonstrate that CMV Cre NoGlow animals provide robust tolerance to each component of the construct (i.e., GFP, Luc, and rtTA3), and enable dynamic tracking and genetic regulation of transplanted tumor lines.

### Tolerant NoGlow Animals Reveal Metastatic Dynamics not Appreciated in Nontolerant Mice

**FIGURE 6:**
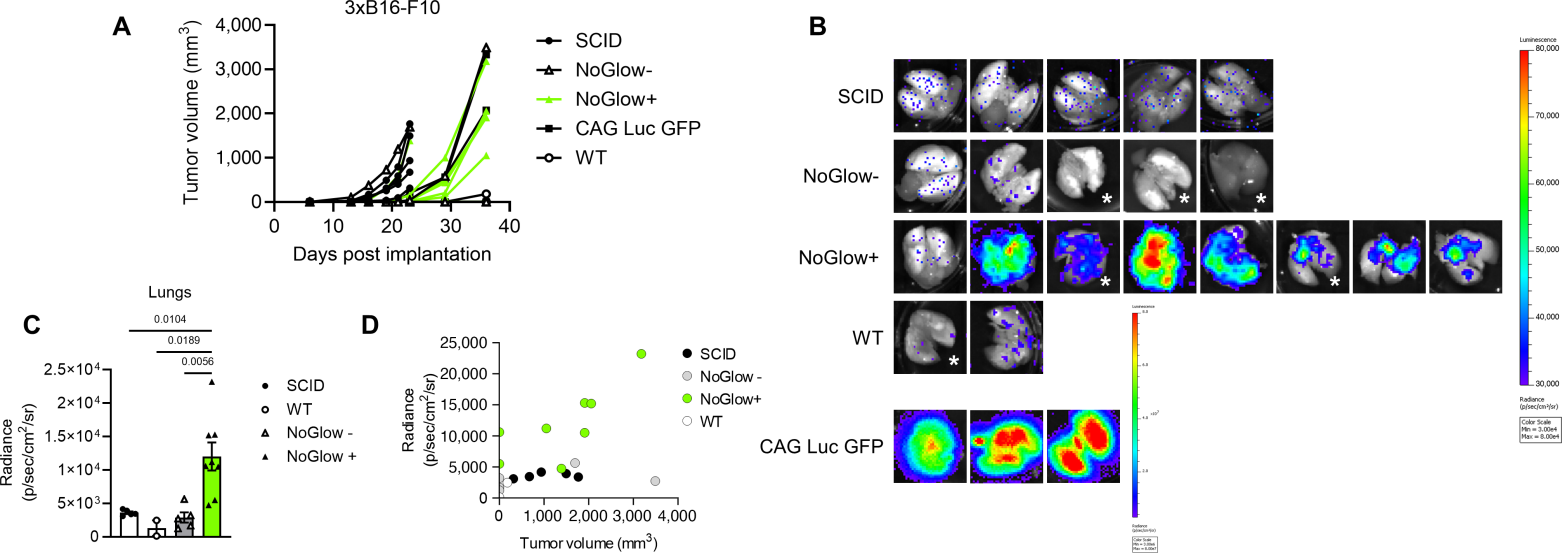
NoGlow mice reveal unappreciated metastatic dynamics independent of the primary tumor. **A,** Tumor growth of triple transgenic (3 ×) B16-F10 cells (10^5^) after subcutaneous implantation into the flank of WT C57Bl/6 (*n* = 2), CAG-driven Full-body WT GFP/Luc expressing mice (*n* = 3), CMV cre littermates positive (*n* = 8) or negative (*n* = 5) for the NoGlow construct, or SCID beige (*n* = 5) mice. **B,** Bioluminescence imaging of lungs at the time of euthanasia for each mouse from A. Robust signal is detected only in NoGlow+ lungs. *Indicates no detectable primary tumor at the end of experiment. **C,** Bioluminescence intensity of individual lungs from B. NoGlow+ lungs contained significantly more positive Luc signal than SCID, WT, or NoGlow− animals. **D,** Plot of tumor volume at time of euthanasia (x) by total lung radiance (y) for individual animals from A. *P* values were determined by one-way ANOVA with Tukey correction. All *P* values represent mean ± SEM.

**FIGURE 7:**
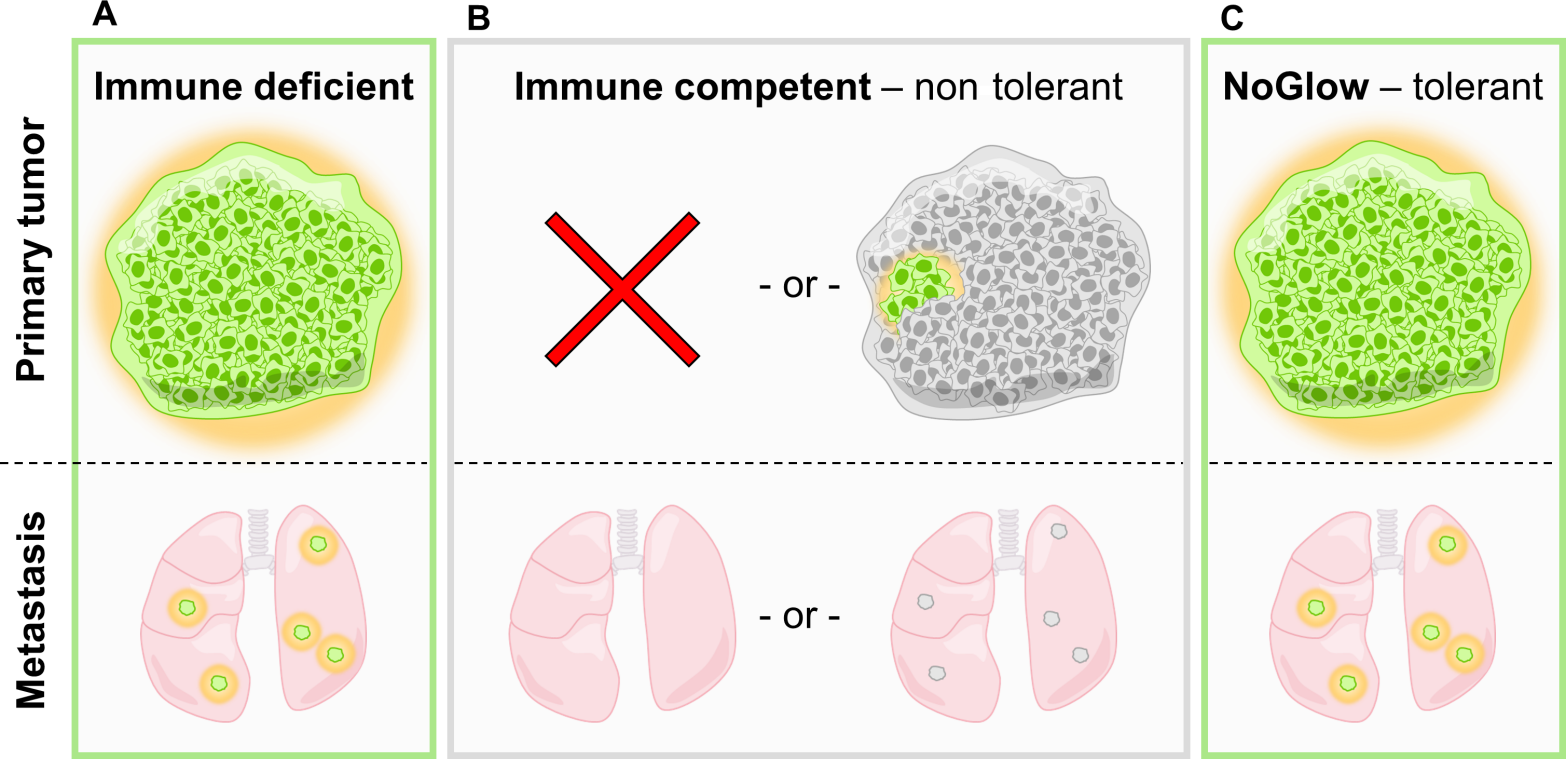
Summary of tumor behavior in differentially tolerant animal models. **A,** Xenoantigen-bearing tumor cells implanted orthotopically into immune-deficient mice typically grow and metastasize as expected and can be easily observed with fluorescence/bioluminescence. **B,** Conversely, in immune-competent animals the presence of foreign immunogens often results in complete rejection of the primary tumor, or severely restricts expression of immunogenic antigens and limits the utility of reporter proteins. **C,** However, NoGlow mice are tolerant to foreign GFP, Luciferase, and rtTA, thus allowing primary tumor growth and *de novo* metastasis of xenoantigen-expressing tumor cells.

To ensure that the NoGlow model is broadly applicable for *in vivo* tumor studies, we generated triple-transgenic 3xB16-F10 melanoma cells (syngeneic in C57Bl/6), a melanoma clonal cell line known to readily metastasize (35). These modified B16-F10 cells (10^5^) were implanted subcutaneously into the flank of male mice and Luc expression was induced on day 23. By the end of the experiment, some 3xB16-F10 tumors formed in all genotypes tested (Fig. 6A). However, of immune-competent animals, heterozygous NoGlow+ animals formed tumors at the injection site most commonly (6/8), followed by CAG Luc GFP (2/3), WT (1/2), and NoGlow- littermates (2/5). In contrast, at the time of euthanasia only NoGlow+ animals harbored robust lung metastasis observable via bioluminescence imaging (Fig. 6B and C). Surprisingly, bioluminescence was even detected in NoGlow+ animals without tumors at the primary site. Furthermore, primary tumor-bearing NoGlow+ mice contained higher metastatic burdens in the lungs than SCID mice with similar tumor volumes (Fig. 6D). These data suggest that in more immune tolerant mice, growth at the primary site does not does always predict metastatic seeding of distant sites. Importantly, use of nontolerant animals precludes this nuanced phenotype while tolerant NoGlow+ mice permit a robust, clinically relevant view of tumor progression (Fig. 7).

## Discussion

Genetic modification, whether of syngeneic, transplantable tumor cell lines or genetically engineered animal models, is an essential tool for basic and translational research. Unfortunately, foreign proteins used for gene regulation and/or cellular tracking add a significant immunologic toll that substantially limits the utility of these tools in vivo. Here we demonstrate that the NoGlow model mitigates many of the caveats in traditional animal models and allows for robust engraftment of transgenic, transplantable tumor cells expressing xenobiotic antigens (i.e., rtTA/GFP/Luc). Importantly, these mice enable *de novo* metastasis and visualization in the lungs of transgenic tumor cells after subcutaneous injection into the flank or orthotopic implantation in the MFP. Thus, this model will be useful to study the full range of tumor–immune interactions during the metastatic cascade without concern of “off-target” immune-based elimination.

Others have attempted to centrally tolerize mice to enable robust tumor engraftment, such as the GH mouse described earlier (11). In a recent study, Grzelak and colleagues used Cx3cr1-Cre or AIRE-Cre animals to induce central tolerance to GFP, however background native GFP fluorescence was not addressed (5). Mice with nonfunctional versions of trackable markers have also been developed; however, these models do not solve the significant effect of tissue-specific tolerance we outline herein. In one report, Bresser and colleagues generated a mouse with constitutive expression of a large transgene encoding different portions of multiple fluorescent genes to elicit whole-body tolerance (36). While an improvement over naïve models, this model focused mostly on fluorophores and unintentionally generated multiple unique junctional epitopes without expression of full-length versions of these proteins. As such, this model may be sufficient for studies of T-cell tolerance but may not be ideal for studies requiring B-cell tolerance that necessitate intact antigens. Therefore, although these models utilized different strategies to elicit immune tolerance, they still contain native function of trackable markers, alter antigens in ways that may impact immune tolerance, or critically lack organ-specific or temporally-inducible expression of the transgenic allele to determine the effect on immunity.

The role of the adaptive immune system, and by extension the effectiveness of targeting this aspect of tumor biology, is now well established. However, both the successes and failures of immunotherapies in the clinic highlight critical gaps in understanding of many aspects of anti-tumor immunity, especially in advanced and metastatic stages. We recently showed that dormant tumor cells resist xenoantigen-specific adaptive immunity – despite high MHC-I – but proliferative counterparts are rejected when expressing foreign genes in immune-competent hosts (18). Thus, immune-competent preclinical models that allow engraftment of the “immune-sensitive” populations are necessary to decipher adaptive immune interactions to a heterogeneous tumor and examine mechanisms of immune-evasion that develop over time. Further still, preliminary investigations using NoGlow mice suggest that the presence of the adaptive immune system can yield increased metastasis, which raises the intriguing possibility that the adaptive immune system promotes metastasis in certain conditions, as has been observed previously (37, 38). For instance, antigen-specific regulatory T cells, which would be compromised in a nontolerant mouse, are a fundamental component of T-cell tolerance and are suggested to influence the processes of autoimmunity and antitumor immunity, as well as promote tumor cell metastasis (39–42). We also show here that sudden exposure to foreign antigens after the window of central tolerance yields a robust immune response to those antigens. In this case, NoGlow mice may provide benefit in endogenous tumor models that are initiated at later time points. *In vivo* screens, which rely on foreign proteins such as Cas9, may also be particularly affected by this limitation. In the future, we anticipate incorporating this and other genes into the NoGlow model for broadly tolerant animals.

Likewise, we observed varying, but complimentary, impacts on tumor progression when testing different tumor cell lines in the NoGlow model. As mentioned, in our hands the mammary tumor line E0771 is highly immunogenic which results in occasional rejection of even parental cells (19). In contrast, syngeneic B16-F10 melanoma cells are reported to be poorly immunogenic and resist immune checkpoint blockade (43, 44). Indeed, these dynamics were repeated in our studies as, other than immune-deficient SCID, triple-transgenic E0771 cells only produced orthotopic tumors in tolerant NoGlow+ mice. Conversely, triple-transgenic B16-F10 melanoma cells were more resistant to rejection in the flank and were able to generate primary tumors in a minority of non-tolerant hosts. However, robust metastases in the lungs of tumor-bearing mice were only observed in NoGlow+ animals. Intriguingly, bioluminescent metastases were detected in lungs of NoGlow+ animals that had undetectable primary tumor growth, suggesting that metastatic seeding occurred early and tumor cells were protected from immune attack in the metastatic niche in NoGlow+ mice (45, 46). These results imply that immune tolerance to xenoantigens permits or even promotes de novo metastasis of implanted tumors, reflecting the ability for many cancers to metastasize independent of primary tumor growth (45, 47, 48).

While crossing the NoGlow model to a variety of tissue-restricted Cre models confirmed their general applicability, we still observed a significant impact of tissue-specific expression on overall tolerance. Whether this difference is mediated by central versus peripheral tolerance, including thymic expression of the NoGlow allele is yet to be determined. Tissue-specific peripheral tolerance, and preventing aberrant autoimmunity, is an important consideration for therapies targeting antigens shared by tumor and normal cells. Intriguingly, multiple rounds of vaccination with self-antigens could break immune tolerance, enhance avidity maturation, and manage tumor growth seemingly without severe autoimmunity in an intestinal fatty acid-binding protein (iFABP)-Ova model (49). If this is true for other tissues, especially when tumor and normal cells sharing the antigen are in close proximity (as with HER2 in the mammary gland), is an important clinical question that remains unanswered but could be addressed in our model. Overall, the NoGlow mouse provides a robust system to study the immune system in the context of oncology as well as basic and translational questions of autoimmunity, immune privilege, gene therapy, and transplantation.

## Supplementary Material

Figure S1 Additional E0771 tumor growth in WT, GH, and CAG Luc-GFP mice; MMTV CAG HER2 Ad-HER2 vaccination T cell responses.

Figure S2 Validating mutant GFP and Luc activity in Adenovirus vaccines

Figure S3 Validating the NoGlow construct in vitro

Figure S4 Confirming triple-transgenic GFP rtTA-Luc construct in E0771 cells

Table S1 Description of animal models used in the study

Table S2 Peptides used for ELISPOTs
