# Supplementary material for: Overcoming Xenoantigen Immunity to Enable Cellular Tracking and Gene Regulation with Immune-competent “NoGlow” Mice
Source: Cancer Res Commun. 2024 Apr 9;4(4):1050–62. doi: 10.1158/2767-9764.CRC-24-0062 (PMC11003454; doi:10.1158/2767-9764.CRC-24-0062)
Supplement: Figure S1 — Additional E0771 tumor growth in WT, GH, and CAG Luc-GFP mice; MMTV CAG HER2 Ad-HER2 vaccination T cell responses. [file crc-24-0062-s01.pdf]

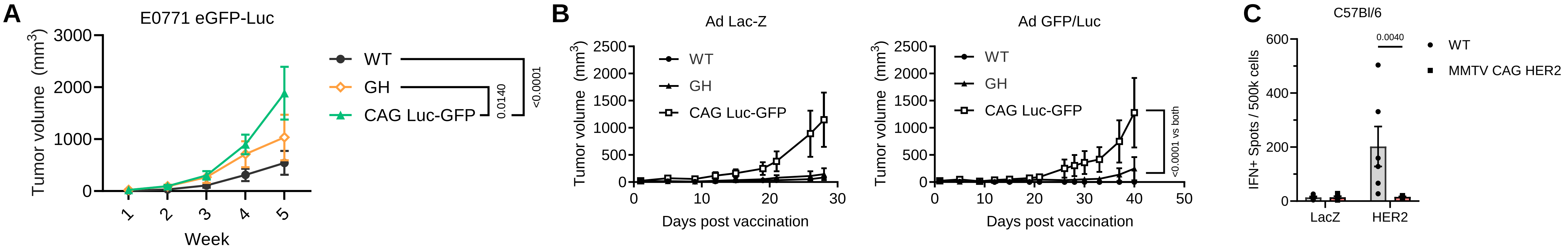

Supplementary Figure 1: **A**) Tumor growth after E0771 eGFP-Luc cells (10^6) were implanted into the MFP of wild-type (WT) C57Bl/6, Glowing Head (GH) or full-body CAG Luc-GFP mice (n=5 each). **B**) Tumor growth plots related to Figure 1C,D. P values for A, B are by 2-way ANOVA with Tukey's multiple comparisons test at final measurement. **C**) C57Bl/6 MMTV CAG HER2 or WT littermates (n=6 each) were vaccinated with Ad-Human HER2 and euthanized after 3 weeks for anti-HER2 T cell responses. All P values represent mean ± SEM.
