# Supplementary material for: Overcoming Xenoantigen Immunity to Enable Cellular Tracking and Gene Regulation with Immune-competent “NoGlow” Mice
Source: Cancer Res Commun. 2024 Apr 9;4(4):1050–62. doi: 10.1158/2767-9764.CRC-24-0062 (PMC11003454; doi:10.1158/2767-9764.CRC-24-0062)
Supplement: Figure S2 — Validating mutant GFP and Luc activity in Adenovirus vaccines [file crc-24-0062-s02.pdf]

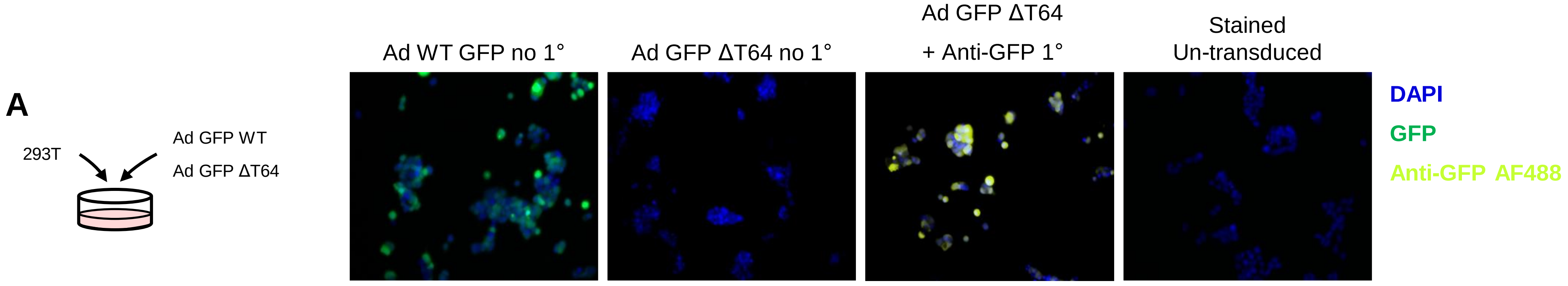

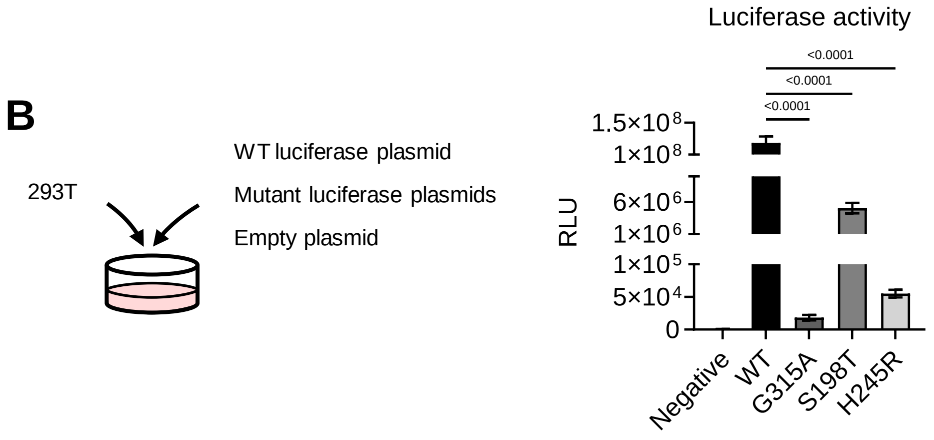

Supplementary Figure 2: A) 293T cells were plated on chamber slides and adenovirus encoding WT or mutant GFP (ΔT64) was added. Cells were fixed, stained for GFP, and imaged for native GFP fluorescence or conjugated fluorescent antibody. **B**) 293T cells were transfected in quadruplicate with WT or the indicated mutant luciferase plasmids and luciferase activity was measured after 48 hours.
