# Supplementary material for: Overcoming Xenoantigen Immunity to Enable Cellular Tracking and Gene Regulation with Immune-competent “NoGlow” Mice
Source: Cancer Res Commun. 2024 Apr 9;4(4):1050–62. doi: 10.1158/2767-9764.CRC-24-0062 (PMC11003454; doi:10.1158/2767-9764.CRC-24-0062)
Supplement: Figure S3 — Validating the NoGlow construct in vitro [file crc-24-0062-s03.pdf]

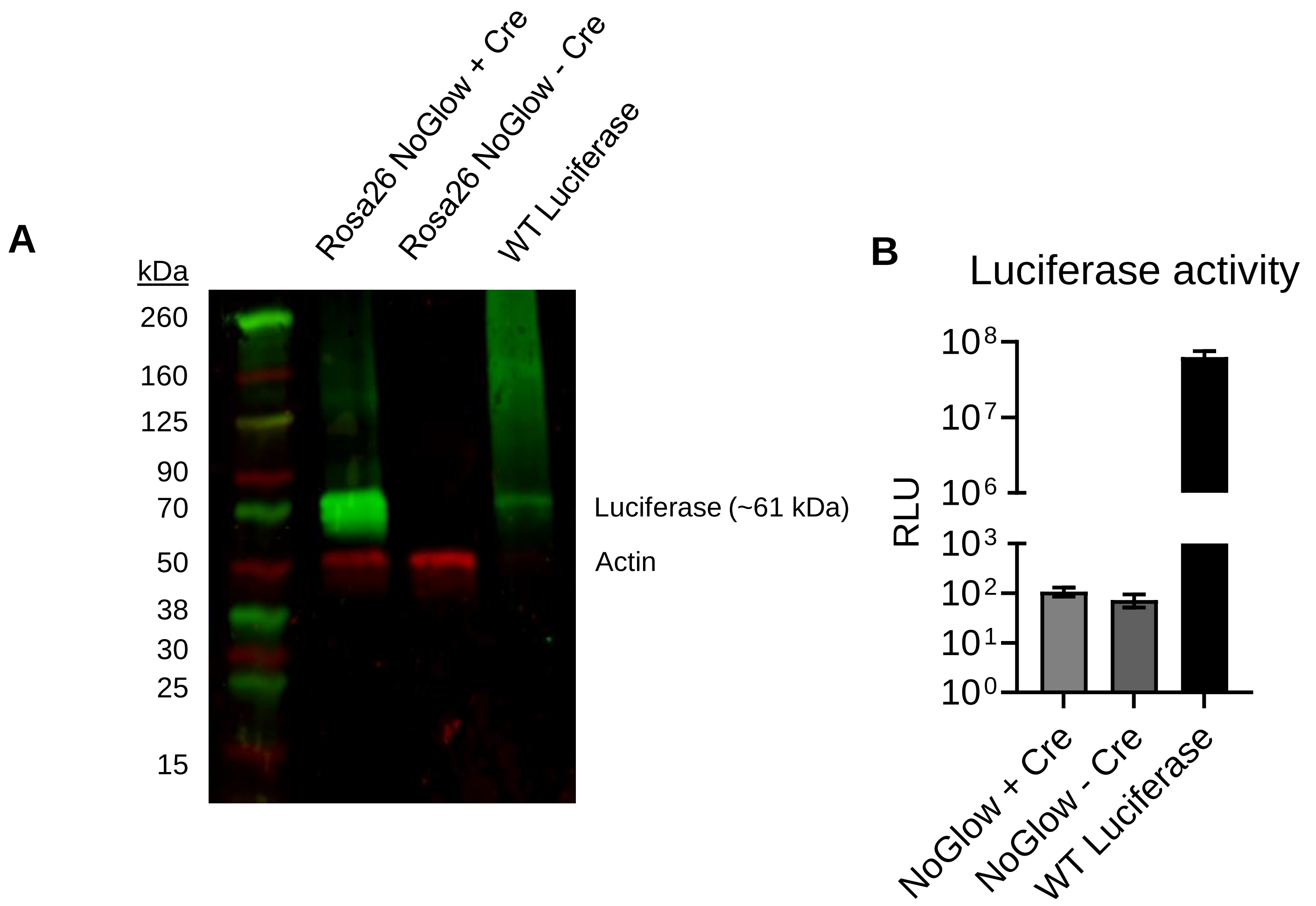

Supplementary Figure 3: A) 293T cells were transfected with plasmid containing the NoGlow construct with or withought co-transfection of plasmid containing Cre recombinase. Western blot for luciferase protein demonstrating Cre-mediated luciferase expression. **B**) Luciferase activity of the same sample from A.
