# Supplementary material for: Overcoming Xenoantigen Immunity to Enable Cellular Tracking and Gene Regulation with Immune-competent “NoGlow” Mice
Source: Cancer Res Commun. 2024 Apr 9;4(4):1050–62. doi: 10.1158/2767-9764.CRC-24-0062 (PMC11003454; doi:10.1158/2767-9764.CRC-24-0062)
Supplement: Figure S4 — Confirming triple-transgenic GFP rtTA-Luc construct in E0771 cells [file crc-24-0062-s04.pdf]

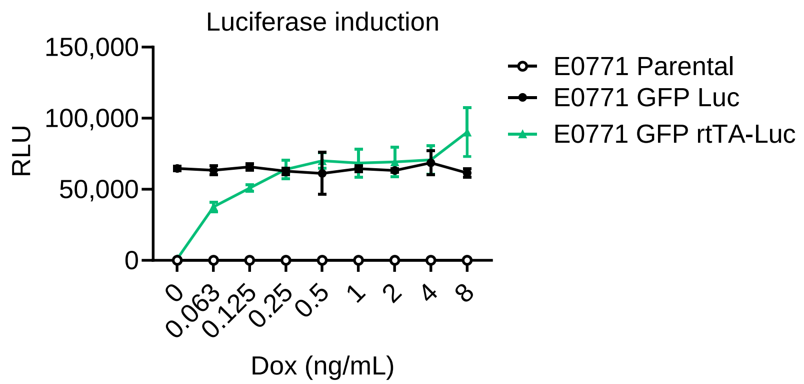

Supplementary Figure 4: Triple-transgenic (3×) E0771 cells constitutively express GFP and rtTA expressed and Luc is induced with the addition of doxycycline in vitro.
