# Supplementary material for: Overcoming Xenoantigen Immunity to Enable Cellular Tracking and Gene Regulation with Immune-competent “NoGlow” Mice
Source: Cancer Res Commun. 2024 Apr 9;4(4):1050–62. doi: 10.1158/2767-9764.CRC-24-0062 (PMC11003454; doi:10.1158/2767-9764.CRC-24-0062)
Supplement: Table S1 — Description of animal models used in the study [file crc-24-0062-s05.pdf]

Strain	Strain detail	Obtained from	Catalog # (if available)	Model description	Reference
SCID-beige	C.B-Igh-1b/GbmsTac-Prkdcscid-Lystbg N7	Taconic Biosciences	CBSCBG	Contains the scid mutation resulting in V(D)J rearrangement defects and lack of B and T lymphocytes. Also carries the beige (Lyst bg) mutation which results in cytotoxic T and macrophage defects as well as NK cell impairment.	
Balb/c	BALB/cAnNTac	Taconic Biosciences	BALB	Standard Balb/c strain	
Glowing Head	C57BL/6-Tyrc-Brd Tg(Gh1-luc/EGFP)D8Mrln/J	Jackson Laboratory	027662	Express a Luc-eGFP fusion gene under the rat growth hormone (Gh1) promoter targeted to the anterior pituitary.	
CAG Luc-GFP	B6;FVB-Ptprca Tg(CAG-luc,-GFP)L2G85Chco Thy1a/J	Jackson Laboratory	025854	Widely express Luc and eGFP directed by the CAG promoter.	
Doxycycline Inducible GFP	B6;129S4-Gt(ROSA)26Sortm1(rtTA*M2)Jae Col1a1tm7(tetO-HIST1H2BJ/GFP)Jae/J	Jackson Laboratory	016836	Express tetracycline-dependent minimal CMV promoter-driven eGFP downstream of the mouse *Col1a1* locus in addition to rtTA in the ROSA locus. Upon doxycycline induction widespread expression of eGFP is observed.	
FoxP3-GFP	B6.129(Cg)-Foxp3tm3(DTR/GFP)Ayr/J	Jackson Laboratory	016958	Express the human diptheria toxin receptor and eGFP downstream of the endogenous *Foxp3* locus. In the abence of diphtheria toxin mice are used as T regulator cell reporters.	
CMV Cre	B6.C-Tg(CMV-cre)1Cgn/J	Jackson Laboratory	006054	Deletes loxP-flanked regions in all tissues.	
MMTV Cre	Tg(MMTV-cre)4Mam/J	Jackson Laboratory	003553	Contain a MMTV LTR promoter driven Cre for deletion of loxP-flanked regions in the virgin and lactating mammary gland.	
Pdx1 Cre	B6.FVB-Tg(Pdx1-cre)6Tuv/J	Jackson Laboratory	014647	Express Cre under the mouse Pdx1 promoter for pancreas-specific deltion of lopP-flanked regions.	
PBSN Cre	FVB;B6-Tg(Pbsn-cre)20Fwan/J	Jackson Laboratory	023325	Express Cre under the composite rat probasin (Pbsn) promoter for prostate-targeted deltion of loxP-flanked regions.	
WAP Her2	B6.Cg-Pds5bTg(Wap-ERBB2)229Wzw/J	Wei-Zen Wei (Wayne State University)	Jackson Labs 010562	Express human HER2 under the control of the mouse whey acid protein promoter (Wap) for expression in the mammary gland.	PMID: 14634087
MMTV CAG-HER2		Joshua Snyder (Duke University)		Contain Cre inducible and CAG driven fluorescently barcoded human HER2 isoforms (Wild type, HER2 d16, HER2 p95) in addition to MMTV Cre for expression in mammary epithelium.	PMID: 34131071

Supplementary Table 1: Animal strains used throughout the study.
