# Supplementary material for: Overcoming Xenoantigen Immunity to Enable Cellular Tracking and Gene Regulation with Immune-competent “NoGlow” Mice
Source: Cancer Res Commun. 2024 Apr 9;4(4):1050–62. doi: 10.1158/2767-9764.CRC-24-0062 (PMC11003454; doi:10.1158/2767-9764.CRC-24-0062)
Supplement: Table S2 — Peptides used for ELISPOTs [file crc-24-0062-s06.pdf]

Peptide	Sequence (N' - C')	Strain	Source
EGFP-C	DTLVNRIEL	C57Bl/6	Genscript
FFL-C	LMYRFEEEL	C57Bl/6	Genscript
LacZ-C	ICPMYARV	C57Bl/6	Genscript
LacZ-B	TPHPARIGL	Balb/c	Genscript
EGFP-B	HYLSTQSAL	Balb/c	Genscript
FFL-B	GFQSMYTFV	Balb/c	Genscript
Human HER2	Peptide pool		JPT
HBV	Peptide pool		JPT

Supplementary Table 2: Peptides used for ELISPOTs.
